# The Impact of Different CD4 Cell-Count Monitoring and Switching Strategies on Mortality in HIV-Infected African Adults on Antiretroviral Therapy: An Application of Dynamic Marginal Structural Models

**DOI:** 10.1093/aje/kwv083

**Published:** 2015-08-26

**Authors:** Deborah Ford, James M. Robins, Maya L. Petersen, Diana M. Gibb, Charles F. Gilks, Peter Mugyenyi, Heiner Grosskurth, James Hakim, Elly Katabira, Abdel G. Babiker, A. Sarah Walker

**Keywords:** Africa, antiretroviral therapy, drug switching, dynamic marginal structural models, HIV, monitoring

## Abstract

In Africa, antiretroviral therapy (ART) is delivered with limited laboratory monitoring, often none. In 2003–2004, investigators in the Development of Antiretroviral Therapy in Africa (DART) Trial randomized persons initiating ART in Uganda and Zimbabwe to either laboratory and clinical monitoring (LCM) or clinically driven monitoring (CDM). CD4 cell counts were measured every 12 weeks in both groups but were only returned to treating clinicians for management in the LCM group. Follow-up continued through 2008. In observational analyses, dynamic marginal structural models on pooled randomized groups were used to estimate survival under different monitoring-frequency and clinical/immunological switching strategies. Assumptions included no direct effect of randomized group on mortality or confounders and no unmeasured confounders which influenced treatment switch and mortality or treatment switch and time-dependent covariates. After 48 weeks of first-line ART, 2,946 individuals contributed 11,351 person-years of follow-up, 625 switches, and 179 deaths. The estimated survival probability after a further 240 weeks for post-48-week switch at the first CD4 cell count less than 100 cells/mm^3^ or non-*Candida* World Health Organization stage 4 event (with CD4 count <250) was 0.96 (95% confidence interval (CI): 0.94, 0.97) with 12-weekly CD4 testing, 0.96 (95% CI: 0.95, 0.97) with 24-weekly CD4 testing, 0.95 (95% CI: 0.93, 0.96) with a single CD4 test at 48 weeks (baseline), and 0.92 (95% CI: 0.91, 0.94) with no CD4 testing. Comparing randomized groups by 48-week CD4 count, the mortality risk associated with CDM versus LCM was greater in persons with CD4 counts of <100 (hazard ratio = 2.4, 95% CI: 1.3, 4.3) than in those with CD4 counts of ≥100 (hazard ratio = 1.1, 95% CI: 0.8, 1.7; interaction *P* = 0.04). These findings support a benefit from identifying patients immunologically failing first-line ART at 48 weeks.

In high-income settings, human immunodeficiency virus (HIV)-positive patients on antiretroviral therapy (ART) receive individualized care. Treating clinicians use routine plasma HIV viral-load measurements and CD4 cell counts every 3–6 months to monitor the efficacy of personalized initial regimens and to trigger ART changes. In Africa, most HIV patients receive ART through the public sector, on the basis of standardized first-line treatment regimens, with little laboratory monitoring to identify failure and trigger switches to second-line ART (1). In the Development of Antiretroviral Therapy in Africa (DART) Trial, investigators randomized 3,316 Ugandan and Zimbabwean adults to receipt of either laboratory and clinical monitoring (LCM), including 12-weekly CD4 cell counts, or clinically driven monitoring (CDM), where CD4 counts were measured but results were not returned to treating clinicians (2). Comparing randomized groups over a median of 4.9 years of follow-up, there was a small but significant survival difference at 5 years (90% for the LCM group vs. 87% for the CDM group), but at current costs, 12-weekly CD4 monitoring was not cost-effective in Uganda/Zimbabwe (3). Testing CD4 cell count less frequently than every 12 weeks would reduce costs, but this was not evaluated in a randomized comparison. The DART data are ideal for observational analyses of different CD4 cell-count monitoring frequencies because all participants had CD4 counts performed but switching was variable following low CD4 counts—firstly by design, since clinicians did not receive test results in the CDM group and the LCM CD4 switch threshold changed during the trial, and secondly because, although compliance was high, it was not complete.

We therefore combined DART randomized groups and estimated survival under different monitoring-frequency and switching strategies after 48 weeks on ART. We first used dynamic marginal structural models and inverse weighting (4–6) to compare survival under switching at the first CD4 cell count below a threshold of 10–100 cells/mm^3^ or the first nonesophageal *Candida* World Health Organization (WHO) stage 4 event (7) (provided that CD4 count was <250 cells/mm^3^ (8)) versus switching for the first WHO 4 event alone. Dynamic marginal structural models have previously been used to estimate “when to start” ART (9). A “when to switch” application is similar (6). Loosely, survival is estimated for each switching strategy, censoring individuals if they become “noncompliant” with the strategy, using weights to account for censoring. We further estimated survival for CD4-count monitoring frequencies ranging from every 12 weeks (12-weekly) to a single CD4 measurement. The same methodology can be utilized, provided that the CD4 test itself (as opposed to the result) has no biological effect on survival (10). An individual's “compliance” with a strategy (e.g., switch at first CD4 count <100 where CD4 counts are measured at baseline and 48-weekly) then depends on the CD4 counts which would have been observed under the strategy (at 0, 48, 96, 144, … weeks).

## METHODS

At DART enrollment in 2003–2004, ART-naive Ugandan/Zimbabwean adults initiated triple-drug ART (zidovudine/lamivudine plus abacavir, tenofovir, or nevirapine) (2). Participants visited the study clinic every 4 weeks (>98% attendance), when nurses administered standard symptom and adherence checklists and prescriptions were dispensed. Participants saw a physician and underwent lymphocyte subset and hematology/biochemistry testing at weeks 4 and 12 and then 12-weekly. All LCM results were returned to clinicians, whereas CDM hematology/biochemistry results were returned only if requested for clinical reasons or if there was grade 4 toxicity; CDM lymphocyte subsets were never returned. Nurses could refer participants to a physician at any time.

Following WHO guidelines (11), a switch to second-line ART (with a ritonavir-boosted protease inhibitor) was discouraged before 48 weeks. The switch decision was based on clinical failure criteria (a WHO 4 event, or a WHO 3 event at the physician's discretion, particularly if recurrent/persistent) in both groups and immunological criteria (CD4 cell count <50 cells/mm^3^ or a confirmed CD4 count <100 cells/mm^3^ from July 2006 onward) in the LCM group (not the CDM group). LCM participants with a low CD4 count could have a repeat CD4 count at/before their next nurse visit. Within-class antiretroviral drug substitutions for adverse events/drug-drug interactions were not considered treatment switches.

### Statistical methods

Study entry was the first 4-week visit at/after 48 consecutive weeks on first-line ART (allowing interruptions of <31 days, usually because of inability to visit the clinic). Follow-up ended at death, December 31, 2008 (trial closure), or the last clinic visit for persons lost to follow-up. Individuals were only classified as lost if, after clinic nonattendance, active tracing through 3 home visits failed. Follow-up data were organized into 4-weekly intervals, beginning 0, 28, 56,  …days after baseline, corresponding to the nurse visit schedule.

Dynamic marginal structural models were used to estimate survival under different hypothetical CD4 monitoring-frequency and switching strategies (5, 10). Switching strategies were defined by current CD4 count dropping below a certain threshold and/or occurrence of a WHO 4 event (or a second WHO 3 event for some strategies). Because other DART analyses showed that a CD4 “tie-breaker” at a <250-cells/mm^3^ threshold would reduce unnecessary second-line switches with viral load less than 400 copies/mL (8), WHO 3/4 events were used to define switching strategies only if the last prior CD4 cell count was less than 250. To compare strategies *X* = 1, 2,  …, *n*, we created *n* copies of each individual's data. A participant was first eligible for a second-line switch under strategy *X* in the first 4-week interval after his or her CD4 cell count dropped below the strategy threshold or within the same interval as the WHO event occurred, provided that the event occurred strictly before the switch. A grace period of three 4-week intervals was permitted for switching: the first interval in which the participant was eligible to switch and the following two 4-week intervals—a period covering 1 scheduled physician visit. In practice, this meant that participants were allowed 12–16 weeks to switch regimens following a low CD4 cell count but 8–12 weeks to switch following WHO events, depending on when the event occurred in the first interval. For each strategy *X*, participants were “artificially” censored when their data became incompatible with *X*, either from switching before becoming eligible or from not switching by the end of the grace period after becoming eligible. To adjust for potential bias due to artificial censoring, we applied patient-time–specific weights equivalent to the inverse of the estimated probability of remaining uncensored (following strategy *X*) conditional on covariate history.

We additionally censored a subgroup of participants randomized to 12-week cycles of structured treatment interruptions (STIs) 52 or 76 weeks after ART initiation in a DART substudy (12). Participants randomized to receive continuous therapy in the same substudy were upweighted, with weights dependent on study center and LCM/CDM status; weights were approximately 2, so that persons randomized to receive continuous therapy represented participants on STIs after censoring in addition to themselves, assuming comparability at randomization. To adjust for potential selection bias from loss to follow-up, lost-to-follow-up weights were also estimated using the factors included in the switching weights and any previous switch to second-line ART (13). The time-dependent product of switching, substudy, and lost-to-follow-up weights was used to weight outcome models. Weights were truncated at 10 (9).

**Figure 1.**
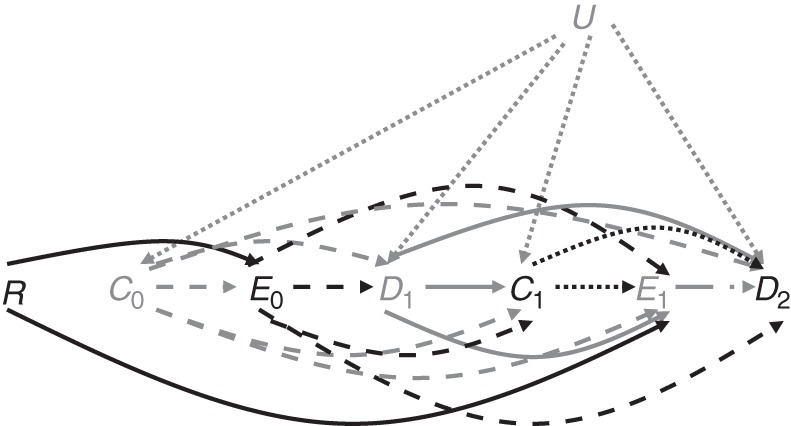
Directced acyclic graph illustrating associations between randomized trial group (*R*), time-dependent covariates at time *t* (*C_t_*, e.g., CD4 cell count), switch to second-line antiretroviral therapy (ART) before/at time *t* (*E_t_*), death before/at time *t* (*D_t_*), and unmeasured common causes of *C* and *D* (*U*) among human immunodeficiency virus–positive patients on ART. Arrows represent direct causal relationships between variables. Time-dependent covariates (*C*) at a given time point influence whether treatment is switched to second-line ART at that time point or subsequently (*E*) and influence time-dependent covariates (*C*) at later time points and mortality (*D*). Switching treatment regimens (*E*) influences time-dependent covariates (*C*), switching (*E*), and mortality at later time points (*D*). The following assumptions are made: *R* has no effect on *C* other than via *E*; *R* has no effect on *D* other than via *E*; there are no unmeasured common causes of *E* and *C* or *E* and *D*; and *R* is randomized. Different line styles and colors are used only to distinguish the effects of randomized group, different covariates, exposures, and death: Effects of *R* are shown by solid black lines; effects of *C*_0_ by dashed gray lines; effects of *E*_0_ by dashed black lines; effects of *D*_1_ by solid gray lines; effects of *C*_1_ by dotted black lines; effects of *E*_1_ by dashed-dotted gray lines; and effects of *U* by dotted gray lines.

Unless otherwise stated, we pooled participants from both randomized groups (LCM/CDM) under the assumptions that 1) participants were comparable at baseline (48 weeks on first-line ART); 2) there was no direct effect of randomized group on mortality or any confounders—that is, any effect on mortality or time-dependent covariates (e.g., CD4 count, WHO 4 events) of access to CD4 test results in the LCM group or lack of access to results in the CDM group was indirect and occured through switching; and 3) there were no unmeasured confounders which influenced treatment switch and mortality or treatment switch and time-dependent covariates (Figure 1). The DART Trial found no difference between randomized groups in any toxicity outcome (2), suggesting a lack of impact of other (non-CD4) laboratory tests. Switching and outcome models were fitted without including randomized group. The rationale for pooling groups and related assumptions are discussed further in Web Appendix 1 (which includes Web Figure 1 and Web Table 1), available at http://aje.oxfordjournals.org/.

The probability of switching to second-line ART during interval *k*, *A*(*k*), where *A*(*k*) = 1 indicates a switch before the end of interval *k*, was estimated using pooled logistic regression:

$$\begin{array}{ll} \text{logit(}P[A(k) & =\text{1|}\bar{A}(k-\text{1)}=\text{0,}\;D(k)=\text{0,}\;V,\;\bar{L}(k)])\\ & =\alpha {}_{0}(k)+{\alpha^{\prime }}_{\;\;2}V+{\alpha^{\prime }}_{\;\;2}\bar{L}(k), \end{array}$$

where α_0_(*k*) is an interval-specific intercept (modeled by restricted cubic splines with knots at the 10th, 50th, and 90th percentiles), $\bar{A}(k-\text{1)}=0$ indicates being on first-line ART up to and including interval *k* − 1, *D*(*k*) = 0 if an individual survived to the end of interval *k*, *V* includes baseline factors at ART initiation and 48 consecutive weeks on first-line ART (analysis baseline), and $\bar{L}(k)$ is history of time-dependent confounders up to interval *k*. Risk factors for mortality and likely confounders were included in the switch model (14). Baseline factors included all possible combinations of study center and first-line ART regimen and the following factors at 48 weeks: CD4 cell count (<100, 100–199, or ≥200 cells/mm^3^), body mass index (weight (kg)/height (m)^2^; ≤18.5, >18.5), hemoglobin concentration (<8 g/dL, ≥8 g/dL), and WHO 4 event 24–48 weeks after starting ART. Time-dependent variables included in $\bar{L}(k)$ were current CD4 cell count (restricted cubic spline with knots at counts of 15, 50, 100, and 200 cells/mm^3^), body mass index (≤18.5, >18.5), and hemoglobin concentration (<8 g/dL, ≥8 g/dL) (for all, the most recent value prior to interval *k* or on the first day of interval *k*), use of cotrimoxazole in the previous interval if this was during the first 72 weeks on ART (15), ≥3 of the previously scheduled 6 nurse visits missed, patient-reported missed ART doses during the 4 weeks prior to interval *k*, and history of WHO stage 3/4 events occurring with CD4 count <250. Body mass index and hemoglobin were categorized because associations were nonlinear. Only the most important predictor (current CD4 count) was modeled using a cubic spline to reduce positivity problems. History of nontuberculosis WHO 3/4 events was included as a 5-category variable with the highest level dominant, as follows: no history in intervals *k* − 5, …, *k*; WHO 3 event or esophageal *Candida* in *k* − 5, …, *k* − 3; WHO 3 event/esophageal *Candida* in *k* − 2, …, *k*; non-*Candida* WHO 4 event in *k* − 5, …, *k* − 3; or non-*Candida* WHO 4 event in *k* − 2, …, *k*. WHO stage 3/4 events in the current interval *k* were included provided that they strictly preceded the switch, since some participants returned to the clinic between visits when sick and had their regimens switched; excluding events occurring in interval *k* was thus likely to introduce unmeasured confounding. An interaction between non-*Candida* WHO 4 events in interval *k* − 2, …, *k* and current CD4 count (<100, ≥100) was included. We considered esophageal *Candida* separately from other WHO 4 events because it is nonfatal and is widely recognized as less severe (16). History of tuberculosis was considered separately from other WHO 3/4 events since clinicians often delayed a switch to start tuberculosis treatment because of potential drug-drug interactions; a 5-level variable ordered similarly to the one above was included (pulmonary tuberculosis = WHO 3, extrapulmonary = WHO 4). Completeness of 4-weekly nurse visits (98%) and 12-weekly physician visits (99%) was very high. Missing values were imputed by car the most recent observation forward (current CD4 count >12/24 weeks previously for 2%/0.4% of intervals, respectively; other variables were similar). Sensitivity analyses additionally included alternative WHO 3/4 event histories, finer CD4 categories for interactions with event history, and CD4 count and body mass index prior to the current one (results were similar; not shown).

To estimate survival under different CD4 monitoring frequencies, we followed Robins et al. (10) and defined each CD4 count as observed or unobserved, depending on the monitoring frequency (e.g., under 48-weekly monitoring, CD4 counts would be observed at 0, 48, 96, … weeks and unobserved at 12, 24, 36, 60, 72, 84, … weeks). We then defined compatibility with switching strategies from observed CD4 counts only (i.e., to define “compliance” with the switching strategy, we ignored “unobserved” CD4 counts). For example, under 48-weekly CD4 monitoring, CD4 thresholds could only be crossed at 0, 48, 96, … weeks and participants who switched in response to other CD4 counts were censored at the time of the switch. Inverse-probability weights were from a switching model including *all* CD4 counts as was done previously, because to control confounding, weights must depend on the original data, including all measured CD4 counts (10).

We estimated survival under an approximately uniform distribution of switching times across the grace period (5). Nonstabilized weights (*W_x_*(*k*) for strategy *X*) were estimated by

$$W_{x}(k)=\frac{I[C_{x}(k)=\text{0}]}{\prod_{\;j=1}^{k}\left\{\;p_{A}{(\;j)}^{I[\;j<Q_{x}]}\right\}}\prod_{r=0}^{m}\begin{array}{c} \left\{{\left\{\frac{\text{1}-\text{1/(}m+\text{1}-r)}{\;p_{A}(Q_{x}+r)}\right\}}^{I[k\geq Q_{x}+r,\;A(Q_{x}+r)=0]}\right.\;\\ \left.\;\;\times {\left\{\frac{\text{1/(}m+\text{1}-r)}{\text{1}-\;p_{A}(Q_{x}+r)}\right\}}^{I[k\geq Q_{x}+r,\;A(Q_{x}+r-1)=0,\;A(Q_{x}+r)=1]}\right\},\; \end{array}$$

where *I*[·] equals 1 if · is true and 0 otherwise, $\;p_{A}(k)\;\mathrm{equals}\text{1}-(P[A(k)=\text{1|}\bar{A}(k-\text{1)}=\text{0,}\;D(k)=\text{0,}\;V,\;\bar{L}(k)]),$ *C_X_*(*k*) is an artificial censoring indicator such that *C_X_*(*k*) = 0 if an individual remains uncensored to the end of interval *k* under strategy *X*, *Q_x_* is the first interval in which an individual is eligible to switch under strategy *X*, *m* is the number of intervals in the grace period excluding the first one (here, *m* = 2), and *r* indexes the 4-week interval within the grace period (here, interval 0, 1, 2). The denominator of the first component is the probability of not switching before becoming eligible to switch. The denominator of the second component is based on the probability of the observed treatment within the grace period, and the numerator forms the uniform distribution of treatment switches across the grace period.

The effects of switching strategies (indexed by *X*) on mortality were estimated in the expanded data set using weighted pooled logistic regression:

$$\begin{array}{l} \text{logit(}P[D(k+\text{1)}=\text{1|}D(k)=\text{0,}\;C_{x}(k)=\text{0,}\;X,\;CF(k+\text{1)}=\text{0])}\\ \;\;=\theta_{0}(k)+{\theta^{\prime }}_{\;\;\;\;1}h(X)+{\theta^{\prime }}_{\;\;\;\;2}g(X)+{\theta^{\prime }}_{\;\;\;\;3}h(X)k_{m}+{\theta^{\prime }}_{\;\;\;\;4}g(X)k_{m}, \end{array}$$

where *D*(*k*) = 1 if an individual died during interval *k*, *CF*(*k*) = 0 if an individual remained uncensored by loss to follow-up/STI to the end of interval *k*, and $\theta_{0}(k)$ is an interval-specific intercept (restricted cubic spline). Either *h*(*X*) or *g*(*X*) is fitted. *h*(*X*) is a linear function of 10 CD4-based strategies, “switch following first CD4 count < *x* or first non-*Candida* WHO 4,” with *x* ranging from 100 to 10 in 10-cell/mm^3^ drops (*h*(*X*) = 0 for event-only-based strategies). *g*(*X*) is a categorical variable for 2 event-only-based strategies (*g*(*X*) = 0 for CD4-based strategies) (Web Appendix 2). *h*(*X*)*k_m_* and *g*(*X*)*k_m_* are interaction(s) between strategy(s) and follow-up time (where *k_m_* = 1 more than 96 weeks from baseline and 0 otherwise). In sensitivity analyses, we considered a spline function for *h*(*X*), and we reduced the number of strategies from 12 to 4 by considering only 2 options for *x*, CD4 <100 or CD4 <50, and fitting a categorical variable for *h*(*X*). Participants who had not switched prior to interval *k* were assumed to have the same mortality risk in interval *k* irrespective of whether or not they switched during *k*; that is, we modeled the probability of death in interval *k* + 1 using weights computed to the end of interval *k*. Monitoring-frequency strategies were modeled as categorical variables keeping the CD4-based strategy fixed, using the outcome model

$$\begin{array}{l} \text{logit(}P[D(k+\text{1)}=\text{1|}D(k)=\text{0,}\;C_{x}(k)=\text{0,}\;X,\;CF(k+\text{1)}=\text{0])}\\ \;\;=\theta_{0}(k)+{\theta^{\prime }}_{\;\;\;\;1}\;\;f\;(X)+{\theta^{\prime }}_{\;\;\;\;2}\;\;f\;(X)k_{m}, \end{array}$$

where *f*(*X*) is a categorical variable defining CD4-count monitoring frequency and *f*(*X*)*k_m_* is an interaction between CD4-count monitoring frequency and follow-up time. Initially, 6 monitoring strategies were considered: 12-, 24-, 48-, or 96-weekly CD4 cell-count monitoring (all with the first CD4 count at baseline), 1 CD4 count measured at baseline only, and no CD4 monitoring. The benefit of a single CD4 count at baseline or 24, 48, …, 240 weeks postbaseline versus no CD4 monitoring (10 strategies) was estimated in a second model of the same form. The models' predicted values were used to estimate survival, with 95% confidence intervals computed using a nonparametric bootstrap (500 replicates, not depending on randomized group).

## RESULTS

Of the 3,316 DART participants (2), we excluded 137 (4%) entered into a nonrandomized pilot STI study 28 weeks after ART initiation, as well as 8 (3 LCM, 5 CDM) switched to second-line ART, 2 (2 LCM) randomized to STIs, 178 (80 LCM, 98 CDM) who had died, and 45 (21 LCM, 24 CDM) who were lost to follow-up before 48 consecutive weeks on first-line ART.

All 2,946 participants included had pre-ART CD4 cell counts less than 200 cells/mm^3^ (median, 86 cells/mm^3^; Table 1). After 48 consecutive weeks on first-line ART, participants in the LCM (*n* = 1,474) and CDM (*n* = 1,472) groups were similar, with median CD4 cell counts of 201 cells/mm^3^ and 200 cells/mm^3^ respectively; 320 of the 2,946 participants (11%) still had CD4 counts less than 100 cells/mm^3^. Excluding and upweighting follow-up after randomization to STI and continuous therapy, respectively (see Methods), produced 11,351 person-years (1,089 person-years (10%) on second-line ART), including 179 deaths (69 deaths (39%) on second-line ART). A total of 190 participants (6%) were lost to follow-up.

**Table 1.** Characteristics and Follow-up of Included Participants Who Completed 48 Consecutive Weeks on First-Line Antiretroviral Therapy, DART Trial, Uganda and Zimbabwe, 2003–2008

Characteristic	LCM Group (*n* = 1,474)			CDM Group (*n* = 1,472)			All Participants (*n* = 2,946)		
	No.	%	Median (IQR)	No.	%	Median (IQR)	No.	%	Median (IQR)
*ART Initiation (DART Randomization)*									
Age, years			37 (32–42)			37 (32–42)			37 (32–42)
Sex									
Male	506	34		528	36		1,034	35	
Female	968	66		944	64		1,912	65	
World Health Organization disease stage									
2	332	23		292	20		624	21	
3	824	56		836	57		1,660	56	
4	318	22		344	23		662	22	
CD4 cell count, no. of cells/mm^3^			84 (33–138)			86 (31–140)			86 (32–139)
Initial ART regimen									
Combivir + tenofovir	1,072	73		1,081	73		2,153	73	
Combivir + abacavir	259	18		250	17		509	17	
Combivir + nevirapine	143	10		141	10		284	10	
*Baseline*^a^									
Time since ART initiation, weeks									
48	1,442	98		1,450	99		2,892	98	
>48–≤72^b^	17	1		9	1		26	1	
>72^b^	15	1		13	1		28	1	
CD4 cell count, no. of cells/mm^3^			201 (140–282)			200 (140–281)			201 (140–281)
<50	40	3		44	3		84	3	
50–99	117	8		119	8		236	8	
100–199	569	39		565	38		1,134	38	
≥200	748	51		744	51		1,492	51	
*Follow-up After 48 Consecutive Weeks on First-Line ART*^c^									
Person-years of follow-up on first-line ART	5,045			5,216			10,262		
No. of deaths on first-line ART	51			59			110		
No. of participants who switched to second-line ART	340			285			625		
Person-years of follow-up on second-line ART	665			424			1,089		
No. of deaths on second-line ART	24			45			69		

Abbreviations: ART, antiretroviral therapy; CDM, clinically driven monitoring; DART, Development of Antiretroviral Therapy in Africa; IQR, interquartile range; LCM, laboratory and clinical monitoring.

^a^ At 48 consecutive weeks on first-line ART.

^b^ Most commonly due to interruptions for adverse events.

^c^ Participants who had structured treatment interruptions in a randomized substudy (*n* = 405) were censored at the first structured treatment interruption, and those randomized to continuous therapy in the substudy were upweighted (by approximately 2; see Methods). Person-years, deaths, and switches shown here *include* this upweighting. Numbers of deaths upweighted: for persons on first-line ART, 5 in LCM group and 2 in CDM group; for persons on second-line ART, 1 in LCM group and 2 in CDM group. Numbers of switches upweighted: 12 in LCM group, 15 in CDM group.

Predictors of a switch to second-line treatment included CD4 count and history of WHO events; 73% of those switching regimens had current CD4 counts under 100 (43% had CD4 counts <50), and 62% switching with CD4 counts of ≥100 had had a WHO 3/4 event in the previous 12 weeks. Characteristics at treatment switch differed in the LCM and CDM groups, broadly following the protocol (Table 2). Ninety-one percent of those switching with a CD4 count <100 and no recent WHO 3/4 event were LCM participants, whereas 76% of those switching following a recent non-*Candida* WHO 4 event were CDM participants. Although similar numbers of persons in the LCM (448/1,474; 30%) and CDM (468/1,472; 32%) groups became eligible to switch for having a CD4 count less than 100 cells/mm^3^ or a non-*Candida* WHO 4 event (with CD4 <250) (Table 3), 72 out of 448 (16%) LCM participants switched during the grace period as compared with 20 of 468 (4%) CDM participants. Few LCM participants became eligible to switch under a strategy delaying the switch to the first WHO 4 event (63/1,474; 4%) or this or 2 WHO 3 events/esophageal *Candida* (110/1,474; 7%), because LCM participants failing first-line ART switched earlier following low CD4 counts.

**Table 2.** Characteristics of Participants at Switch in Antiretroviral Therapy (ART) Regimen After 48 Consecutive Weeks on First-Line ART, DART Trial, Uganda and Zimbabwe, 2003–2008

CD4 Cell Count and Event Type^a^	LCM Group (*n* = 340^b^)		CDM Group (*n* = 285^b^)		All Participants (*n* = 625^b^)	
	No.	Row %	No.	Row %	No.	Column %
<50 cells/mm^3^						
No event	98	85	17^c^	15	115	18
WHO 3 event or esophageal *Candida*	19	19	80	81	99	16
WHO 4 event	9	17	44	83	53	8
50–99 cells/mm^3^						
No event	116	96	5^c^	4	121	19
WHO 3 event or esophageal *Candida*	23	48	25	52	48	8
WHO 4 event	7	37	12	63	19	3
100–249 cells/mm^3^						
No event	38	76	12^c^	24	50	8
WHO 3 event or esophageal *Candida*	15	39	23	61	38	6
WHO 4 event	11	44	14	56	25	4
≥250 cells/mm^3^						
No event	2	14	12^c^	86	14	2
WHO 3 event or esophageal *Candida*	1	5	20	95	21	3
WHO 4 event	1	5	21	95	22	4

Abbreviations: ART, antiretroviral therapy; CDM, clinically driven monitoring; DART, Development of Antiretroviral Therapy in Africa; LCM, laboratory and clinical monitoring; WHO, World Health Organization.

^a^ A WHO stage 3 or 4 event in the current 4-week interval or one of 2 previous 4-week intervals.

^b^ Participants who had structured treatment interruptions in a randomized substudy (*n* = 405) were censored at the first structured treatment interruption, and those randomized to continuous therapy in the substudy were upweighted (by approximately 2; see Methods). Numbers of switches upweighted: 12 in LCM group, 15 in CDM group.

^c^ CDM participants switched without WHO 3/4 events may have had clinical events which did not meet predefined protocol criteria for WHO 3/4 events.

**Table 3.** Compliance With Different Antiretroviral Treatment Switching and CD4 Cell-Count Monitoring Strategies and Estimated Survival, DART Trial, Uganda and Zimbabwe, 2003–2008

Switching or CD4 Cell-Count Monitoring Strategy	LCM Group				CDM Group				All Participants							
	No. of Persons Switched^a^	No. Eligible to Switch^a^	%^b^	No. of Deaths^c^	No. of Persons Switched^a^	No. Eligible to Switch^a^	%^b^	No. of Deaths^c^	No. of Persons Switched^a^	No. Eligible to Switch^a^	%^b^	No. of Deaths^c^	Survival Probability at 192 Weeks^d^	95% CI	Survival Probability at 240 Weeks^d^	95% CI
*Switching Strategy Recommended in the LCM Group During the Trial (CD4 Counts Measured Every 12 Weeks)*																
CD4 count <50 cells/mm^3^ up to June 30, 2006, and <100 cells/mm^3^ thereafter or non-*Candida* WHO 4 event (CD4 count <250)^e^	211	354	60	60	54	376	14	36	265	730	36	96	0.96	0.94, 0.97	0.95	0.94, 0.97
*CD4 Counts Measured Every 12 Weeks, Switching Strategies Varied*																
CD4 count <100 cells/mm^3^ or non-*Candida* WHO 4 event (CD4 count <250)	72	448	16	47	20	468	4	28	92	916	10	75	0.97	0.95, 0.98	0.96	0.94, 0.97
CD4 count <50 cells/mm^3^ or non-*Candida* WHO 4 event (CD4 count <250)	103	218	47	58	38	305	12	42	141	523	27	100	0.96	0.95, 0.97	0.95	0.94, 0.96
2 WHO 3 events or esophageal *Candida* (CD4 count <250) or non-*Candida* WHO 4 event (CD4 count <250)	45	110	41	58	94	231	41	66	139	341	41	124	0.94	0.93, 0.95	0.93	0.91, 0.94
Non-*Candida* WHO 4 event (CD4 count <250)	25	63	40	57	62	144	43	73	87	207	42	130	0.94	0.92, 0.95	0.92	0.91, 0.94
*Switch at First CD4 Cell Count <100 Cells/mm^3^ or Non-*Candida *WHO Stage 4 Event (CD4 Count <250), CD4 Monitoring Frequency Varied*																
CD4 count measured at baseline^f^ and every 12 weeks thereafter	72	448	16	47	20	468	4	28	92	916	10	75	0.97	0.95, 0.98	0.96	0.94, 0.97
CD4 count measured at baseline^f^ and every 24 weeks thereafter	79	387	20	52	24	425	6	31	103	812	13	83	0.97	0.95, 0.98	0.96	0.95, 0.97
CD4 count measured at baseline^f^ and every 48 weeks thereafter	68	311	22	54	32	378	8	37	100	689	15	91	0.96	0.95, 0.97	0.95	0.93, 0.96
CD4 count measured at baseline^f^ and every 96 weeks thereafter	59	256	23	53	40	337	12	42	99	593	17	95	0.96	0.95, 0.97	0.95	0.93, 0.96
CD4 count measured at baseline only^f^	31	199	16	51	39	260	15	47	70	459	15	98	0.96	0.95, 0.97	0.95	0.93, 0.96

Abbreviations: ART, antiretroviral therapy; CDM, clinically driven monitoring; CI, confidence interval; DART, Development of Antiretroviral Therapy in Africa; LCM, laboratory and clinical monitoring; WHO, World Health Organization.

^a^ Participants who had structured treatment interruptions in a randomized substudy (*n* = 405) were censored at the first structured treatment interruption, and those randomized to continuous therapy in the substudy were upweighted (by approximately 2; see Methods).

^b^ Percentage of participants eligible to switch who switched within the grace period.

^c^ Total number of deaths in participants compatible with strategy at the time of death (with weighting for structured treatment interruptions as described in footnote “a” above).

^d^ Survival from baseline (48 consecutive weeks on first-line ART).

^e^ Recommended CD4-based switching in the LCM group was changed from <50 cells/mm^3^ to <100 cells/mm^3^ in July 2006.

^f^ Baseline was defined as the first 4-week visit at or after 48 consecutive weeks on first-line ART.

Comparing groups as randomized, estimated survival from baseline (48 consecutive weeks on first-line ART) was 95% 192 weeks (3.7 years) later and 94% 240 weeks (4.6 years) later in LCM participants versus 93% and 92%, respectively, in CDM participants (hazard ratio = 1.40, 95% confidence interval (CI): 1.02, 1.92; *P* = 0.04). CD4 <50/WHO 4 events were the recommended switching criteria in the LCM group up to July 2006, and CD4 <100/WHO 4 events were the recommended criteria from July 2006 onward (following WHO (11)); pooling randomized groups, the estimated survival for this strategy was marginally higher than that observed in the LCM group, consistent with small improvements under full protocol compliance. Similarly, the estimated survival for switching at the first WHO 4 event, or the first WHO 4 event/multiple WHO 3 events, was similar to observed survival in the CDM group (Table 3). Under 12-weekly CD4 testing, survival was highest for those switching at CD4 <100 or the first non-*Candida* WHO 4 event (with CD4 <250). Using a linear term for the effect of CD4-based strategies, estimated survival 240 weeks after baseline was 96% (95% CI: 94, 97), 95% (95% CI: 94, 96), and 92% (95% CI: 91, 94) for switching at CD4 <100, CD4 <50, and no CD4 threshold, respectively (each with a non-*Candida* WHO 4 event as above) (Table 3). Benefits from switching at CD4 <100/non-*Candida* WHO 4 were 1.0% (95% CI: 0.0, 1.8) in comparison with CD4 <50/non-*Candida* WHO 4 and 3.5% (95% CI: 1.4, 5.6) in comparison with non-*Candida* WHO 4 events only. Results were similar when we fitted only these 2 CD4-based strategies as categories rather than fitting 10 strategies with a linear effect. Adding switching after 2 WHO 3/esophageal *Candida* events produced little survival improvement in comparison with switching for the first WHO 4 event (0.6%, 95% CI: −0.7, 2.0).

**Figure 2.**
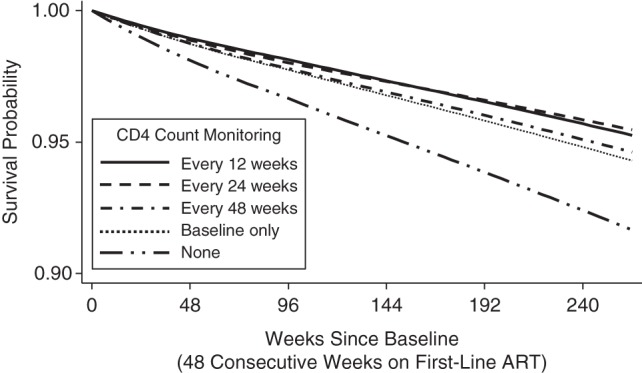
Survival among human immunodeficiency virus–positive patients on antiretroviral therapy (ART) for different CD4 cell-count monitoring strategies, all assuming a switch in treatment regimen (to second-line ART) at the first observed CD4 count less than 100 cells/mm^3^ or the first non-*Candida* World Health Organization stage 4 event (with CD4 count <250), estimated by means of dynamic marginal structural models, Development of Antiretroviral Therapy in Africa (DART) Trial, Uganda and Zimbabwe, 2003–2008. Baseline was 48 consecutive weeks on first-line ART.

Under a strategy defined by switching at the first CD4 count <100 or non-*Candida* WHO 4 event (CD4 <250), we found no survival advantage at 240 weeks for 12-weekly CD4 counts as compared with 24-weekly CD4 counts (−0.2%, 95% CI: −1.4, 0.7) and observed only small, nonsignificant survival advantages for 12-weekly CD4 counts compared with less frequent CD4-monitoring strategies, including a single (baseline) CD4 count 48 weeks after ART initiation (0.9% (95% CI: −1.0, 2.7) at 240 weeks) (Table 3, Figure 2). Compared with no CD4 monitoring, the survival benefit derived from a single CD4 count after 48 weeks of first-line ART was significant (2.4% (95% CI: 1.3, 3.9) at 240 weeks). Under 12-weekly CD4 counts, 2.2% of follow-up would be spent with CD4 count <100 as compared with 2.7% and 3.6% under 24-weekly and 48-weekly CD4 counts, 6.6% with a single CD4 count after 48 weeks of first-line ART, and 9.4% with no CD4 monitoring (Web Figure 2). A single CD4 count after 48 weeks of first-line ART improved survival at 240 weeks by 1.2% (95% CI: 0.2, 2.3) and 1.9% (95% CI: 0.7, 3.5) as compared with a single CD4 count after 72 and 96 weeks of first-line ART, respectively.

In sensitivity analyses, we found similar survival differences across different CD4 testing frequencies when switching criteria were varied to exclude a switch following extrapulmonary tuberculosis, to include a switch at all non-*Candida* WHO 4 events irrespective of current CD4 count, or to depend on event history lagged by 4 weeks (Web Table 2), and irrespective of the use of cotrimoxazole during weeks 48–72 of ART (Web Appendix 3 (including Web Table 3)). Notably, in all sensitivity analyses, a single CD4 count after 48 weeks of first-line ART significantly improved survival 240 weeks later in comparison with no CD4 monitoring: by 2.4% (95% CI: 1.3, 3.6) excluding extrapulmonary tuberculosis, by 2.3% (95% CI: 1.3, 3.5) with no CD4 restriction, and by 1.8% (95% CI: 0.9, 3.1) using lagged events. Using raw rather than truncated weights, the benefits of CD4 monitoring were slightly higher. We also considered a combined outcome including death or loss to follow-up; the percentages of persons alive and under follow-up were 2.4% (95% CI: −0.7, 5.3) and 1.9% (95% CI: 0.3, 3.3) higher for 12-weekly CD4 counts and a single 48-week CD4 count, respectively, in comparison with no CD4 monitoring.

**Figure 3.**
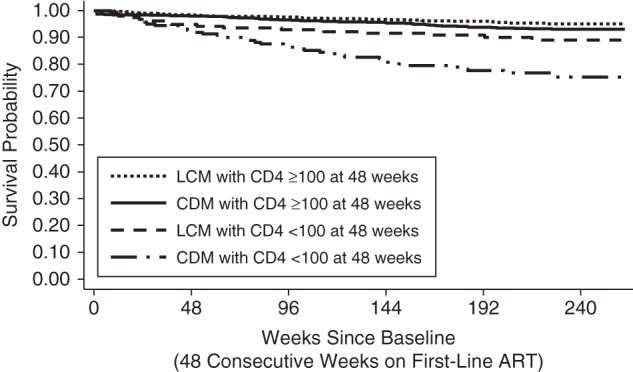
Survival among human immunodeficiency virus–positive patients on antiretroviral therapy (ART), by randomized trial group (laboratory and clinical monitoring (LCM) or clinically driven monitoring (CDM)) and CD4 cell count at 48 consecutive weeks on first-line ART (baseline), Development of Antiretroviral Therapy in Africa (DART) Trial, Uganda and Zimbabwe, 2003–2008.

Given the substantial benefits of a single CD4 count suggested by the dynamic marginal structural model, we compared DART randomized groups by CD4 count at 48 consecutive weeks of first-line ART (Figure 3). Fifty-four (30%) of the 179 subsequent deaths occurred among the 11% of participants with CD4 counts less than 100 cells/mm^3^ at 48 weeks—16 in the LCM group versus 38 in the CDM group (hazard ratio = 2.39, 95% CI: 1.32, 4.32). In contrast, there were 59 LCM deaths versus 66 CDM deaths among participants with CD4 cell counts greater than or equal to 100 cells/mm^3^ at 48 weeks (hazard ratio = 1.13, 95% CI: 0.77, 1.65; interaction *P* for heterogeneity = 0.04). Excluding the 320 participants with CD4 counts less than 100 at 48 weeks, only 525 of 2,626 (20%) participants had a CD4 count less than 100 without a prior WHO 4 event during the subsequent 4 years of follow-up; the observed benefit of CD4 monitoring is small at the population level because such persons are in the minority.

## DISCUSSION

We applied dynamic marginal structural models to DART Trial data to estimate survival under different switching strategies among HIV-infected Ugandan/Zimbabwean adults who completed 48 consecutive weeks of first-line ART. Using these observational analysis methods, we found that with 12-weekly CD4 monitoring, switching therapy when CD4 count drops below 100 cells/mm^3^ is associated with a small but significant survival advantage compared with switching at clinical events.

Using observational analyses within a trial “cohort” further enabled us to investigate the impact of decreasing the frequency of CD4 monitoring on ART effectiveness. To our knowledge, this is a novel application of these methods. In DART, treating clinicians had not received CDM CD4 counts during the trial, although they were measured; this led to sufficient variability in switching to allow us to explore the impact of different CD4 monitoring strategies. We found that 24-weekly CD4 cell counts, used in practice in some African countries (17), were not inferior to 12-weekly CD4 counts in terms of mortality. Of note, a *single* CD4 cell count taken 48 weeks after ART initiation, switching regimens if the count was less than 100 cells/mm^3^, offered a significant survival benefit compared with clinical monitoring only. Direct comparison of randomized groups by 48-week CD4 count supported this, showing that the benefit derived from CD4 monitoring was significantly greater among persons with CD4 counts less than 100 at 48 weeks, with little difference between randomized groups in those with higher CD4 counts. These results suggest that in places where limited laboratory facilities are available, 1 CD4 cell count taken approximately 1 year after ART initiation to identify lack of response to first-line ART and trigger a switch in treatment regimen could improve survival.

The question of how frequently routine ART laboratory monitoring should occur has only been assessed in stochastic modeling studies of cost-effectiveness. Only 1 study (18) directly compared 3-monthly monitoring with 6-monthly monitoring, for both CD4 and CD4 + viral load, and found a <1-month gain in life expectancy from 3-monthly monitoring versus 6-monthly monitoring across a range of ART initiation strategies. However, that study used a 50% CD4 decline from peak level to define immunological failure, in contrast to the more sensitive but relatively late CD4 <100 threshold used in DART (19). The question of how much further survival benefits might increase if switching were done earlier at CD4 thresholds greater than or equal to 100 (or based on plasma HIV RNA level) cannot be addressed using DART data, because these strategies were not followed by sufficient numbers of participants. Although further benefits from other WHO immunological failure criteria (less than pre-ART or 50% decline from peak) may be possible, and although other studies have suggested high survival at 5 years if a second-line switch immediately follows WHO immunological failure (20), gains are likely to be small given that in this analysis 96% of those alive after 48 weeks on ART were predicted to survive a further 4.6 years with 24-weekly CD4 monitoring and a switch at <100 cells/mm^3^.

We pooled DART randomized groups in order to explore the effects of different strategies, because within each randomized group switching patterns were similar. This required assuming no direct effect of group on mortality. There was some suggestion of a nonsignificant residual benefit for CDM participants versus LCM participants (Web Appendix 1), which could suggest that clinicians took better care of patients where they had no access to CD4 counts and that confounders were missed or unmeasured; if this were the case, we may have underestimated the added benefit of more frequent routine CD4 monitoring, since estimates of survival under strategies including less frequent/no CD4 monitoring depended heavily on survival in the CDM group. However, because participants were enrolled in a clinical trial, we had systematic and close-to-complete data on use of other medications, visit attendance, ART adherence, and laboratory and clinical measures, and multiple sensitivity analyses incorporating different aspects of these factors did not affect results.

Application of these methods to a realistic scenario more complex than previously considered raised several important methodological issues. Firstly, clinicians often requested a second CD4 cell count rather than switch for a single low CD4 count. Since confirmatory CD4 counts were only available at selected times and in a subset of patients in our study, they could not sensibly be incorporated into a strategy. Secondly, allowing different grace periods for different clinical events would have been more appropriate clinically, because clinicians often delay a switch to second-line ART following a tuberculosis diagnosis (WHO 3/4 event) due to drug interactions between rifampicin and boosted protease inhibitors. This is complicated by the fact that patients may be eligible to switch for more than 1 event within overlapping intervals; so, for example, a patient whose CD4 count drops below 100 may become eligible to switch but then receive a tuberculosis diagnosis within the grace period prior to switching. We considered strategies including and excluding WHO 4 extrapulmonary tuberculosis as a trigger for switching to ensure that our results regarding CD4 monitoring frequency were robust.

In summary, our results demonstrate how data from well-conducted large randomized controlled trials can be exploited using rigorous observational analyses to address clinical questions beyond those originally anticipated. Trial data have several strengths, including typically higher completeness and collection of data on additional items not available within clinical cohorts or available less frequently in interval cohorts (21). As with all observational analyses, we cannot exclude the possibility of bias, but our findings support increasing access to CD4 cell counts for all patients on ART rather than increasing the frequency of routine laboratory monitoring for patients in easy-to-access areas.

## Supplementary Material

Web Material
