# Supplementary material for: The Impact of Different CD4 Cell-Count Monitoring and Switching Strategies on Mortality in HIV-Infected African Adults on Antiretroviral Therapy: An Application of Dynamic Marginal Structural Models
Source: Am J Epidemiol. 2015 Aug 26;182(7):633–43. doi: 10.1093/aje/kwv083 (PMC4581589; doi:10.1093/aje/kwv083)
Supplement: Web Material [file supp_182_7_633__index.html]

The Impact of Different CD4 Cell-Count Monitoring and Switching Strategies on Mortality in HIV-Infected African Adults on Antiretroviral Therapy: An Application of Dynamic Marginal Structural Models — The Impact of Different CD4 Cell-Count Monitoring and Switching Strategies on Mortality in HIV-Infected African Adults on Antiretroviral Therapy: An Application of Dynamic Marginal Structural Models — Web Material 

# The Impact of Different CD4 Cell-Count Monitoring and Switching Strategies on Mortality in HIV-Infected African Adults on Antiretroviral Therapy: An Application of Dynamic Marginal Structural Models

## Web Material

Web Material

- Web Material - Pdf file
