# Supplementary material for: The Impact of Different CD4 Cell-Count Monitoring and Switching Strategies on Mortality in HIV-Infected African Adults on Antiretroviral Therapy: An Application of Dynamic Marginal Structural Models
Source: Am J Epidemiol. 2015 Aug 26;182(7):633–43. doi: 10.1093/aje/kwv083 (PMC4581589; doi:10.1093/aje/kwv083)
Supplement: Web Material [file supp_kwv083_kwv083supp.pdf]

WEB APPENDIX 1

In this appendix, we outline the motivation for pooling the LCM and CDM randomized groups in our analysis without adjustment for randomized group, we discuss the assumptions we made and how we tested them, and we discuss the effects that pooling groups had on the "switching weights."

The rationale for pooling LCM and CDM randomized groups was to allow us to explore the effects of different switching and monitoring strategies. Within a randomized group, patient management was similar, and there was insufficient variability in switching patterns to allow us to estimate survival across all strategies (e.g., in LCM relatively few patients waited until a WHO stage 4 event to switch and in CDM relatively few patients switched at a CD4 threshold without symptoms). The majority of the information came from the LCM group for the strategy: "switch at first CD4 <100/nonesophageal *Candida* WHO 4 event (with CD4 <250) with 12-weekly CD4 monitoring," but as we considered strategies with less frequent CD4 monitoring, increasingly information was also contributed by CDM patients, since switching at a CD4 threshold (when the clinician did not know the CD4 results) was more variable in the CDM group. Assuming all patients were similar at analysis baseline (after 48 consecutive weeks on ART), we argue that when a patient is artificially censored for noncompliance with a given switching strategy, it is immaterial (under assumptions below) whether a similar CDM or a similar LCM patient who has remained compliant is "upweighted" to account for the censoring (effectively conditional on the time-dependent covariates included in the switching weights). The principle of the weighting model is to balance treatment (switching) for predictors of

survival; if there is no biological benefit of testing CD4 (as opposed to biological benefit from the result value or subsequent patient management) and the difference in patient management between randomized groups is only through an earlier switch in LCM (where clinicians have access to CD4 counts), patients on first-line ART with the same history of covariates should have similar underlying mortality risk irrespective of randomized group.

More formally, we have assumed that there is no direct effect of randomized trial group on survival or confounders. We discuss the implications of our assumption by focusing on 3 questions.

1. How would our conclusions be affected if the "no direct effect" assumption were false?

2. We made the strong assumption that randomized trial group had no direct effect on survival or any confounder rather than the weaker assumption of no direct effect on survival. What would have been the consequences had we only imposed the weaker assumption?

3. What is the positivity problem, and to what extent does an analysis that does not adjust for randomized trial group solve the problem?

Question 1: Suppose that in Figure 1, there was a directed arrow from randomized group R to a time-dependent covariate (i.e., to either C_0 or C_1) to U; or to survival (i.e., D_1 or D_2). Then randomized group would have a direct effect on survival, as there would be a sequence of directed arrows from R to D_2. Hence, R would be a common cause of switch

(i.e., E_1 and E_2) and survival. In this case the effect of different monitoring schedules would not be identified from the pooled trial data. We investigated a possible concern in DART, which illustrates the problem. In the DART Trial, clinicians prescribed cotrimoxazole to participants at their discretion. From the trial data, cotrimoxazole has been shown to significantly reduce the risk of mortality during the first 72 weeks on ART (1). If cotrimoxazole were prescribed differently in the two randomized groups—for example, if clinicians were more likely to prescribe it to patients for whom CD4 measurements were not available—then survival for a given CD4 monitoring strategy (e.g., 96-weekly CD4s with switch at first CD4 <100 or first WHO 4 event) would depend on the extent of cotrimoxazole use under that strategy. However, the extent of use would not be estimable from the DART Trial data except for the two monitoring strategies actually studied in the trial. Because this was a real concern in DART, we looked at survival for different monitoring strategies under no cotrimoxazole between 48–72 weeks on ART and under continuous cotrimoxazole use between 48–72 weeks on ART. We were able to demonstrate similar effects of monitoring strategies on survival under both extremes (Web Appendix 3). There may, however, be other ways in which randomized group affected survival.

Question 2: To answer question 2, we consider the causal DAG shown in Web Figure 1.

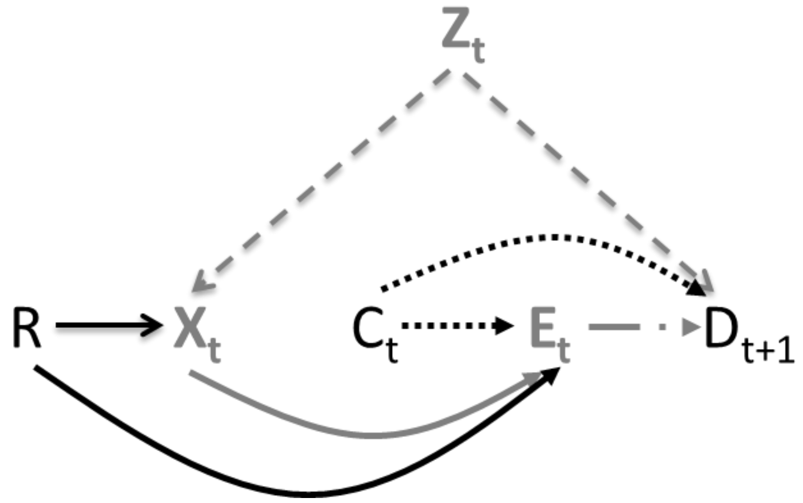

Web Figure 1. Directed acyclic graph (DAG) illustrating associations between randomized group (*R*), time-dependent covariates at time *t* : Z_t, X_t, and C_t , switch to second-line ART before/at time *t* (E_t), and death before/at time *t* + 1 (D_{t+1}). Arrows represent direct causal relationships between variables. Variables and relationships shown in Figure 1 are still assumed, but additionally we have time dependent covariates Z_t and X_t. *Z* influences *X* at that time point and subsequently and mortality at later time points (*D*). *X* influences switching treatment (*E*) at that time point and subsequently, and because randomized group influences *X*, there is now a path from *R* to *D* which does not pass through *E*. Different line styles and colors are used only to distinguish the effects of randomized group, different covariates, exposures and death (e.g., effects of *R* are shown by solid black lines, effects of X_t by solid gray lines).

Here, X_t is a cause of switching but does not have a direct effect on survival. Z_t is a direct cause of death and a cause of X_t. Randomized group (*R*) is a direct cause of the confounder X_t but only affects survival through switching. Nonetheless, *R* must be adjusted for to control confounding. To see this, note, we must condition on X_t to block the backdoor path $E_tX_tZ_tD_{t+1}$, but conditioning on X_t opens the path $E_tRX_tZ_tD_{t+1}$ as X_t is a collider on the path. However, further conditioning on (and thus adjusting for) *R* now renders all backdoor paths blocked. Hence, we are only justified in our decision not to

adjust for *R* under the strong assumption that *R* is not a direct cause of survival or of any confounder.

Question 3: We shall suppose the hypothesis of no direct effect of randomized trial group on survival or any confounder holds, so it is unnecessary to adjust for randomized group. Again, suppose our interest was in the survival curve had all subjects followed the strategy with CD4 monitoring every 96 weeks and with switching the first time the CD4 count was less than 100 or a WHO 4 event was diagnosed. Suppose further that all subjects in both the LCM and CDM groups complied with their assigned protocols (simplified here to be switch at CD4 <100 or first WHO 4 event in LCM and switch at first WHO 4 event in CDM). It is then easy to see and is argued below that if we adjust for randomized group in the analysis, it is not possible to consistently estimate survival under our strategy of interest. We now show that even if, as in the body of the paper, we ignore randomized group in the analysis, we still cannot consistently estimate this survival curve. To understand why, consider a subject in the LCM group whose CD4 count was first found to be below 100 at week 48 and who thus was switched in that week. Since that subject failed to follow the strategy of interest, we must censor him at week 48 and transfer his weight to similar subjects (i.e., subjects with the same past CD4 count history) who remain on the strategy of interest. Now under complete compliance there are no such similar subjects in the LCM group, as all subjects with the same CD4 history would also have switched and thus been censored. Thus, if we had adjusted for (i.e., stratified on) treatment group, we could not consistently estimate survival under our strategy of interest. When no similar uncensored subjects are available to represent a censored subject, we say that there is a violation of positivity. When not adjusting for randomized group, similar uncensored subjects do exist.

The similar uncensored subjects are precisely the subset of the CDM group whose CD4 count first went below 100 at week 48. However, now consider week 96. All members of the CDM group without a WHO 4 event who have a CD4 count under 100 at week 96 will be censored at that time because, under the strategy of interest, they must switch; but because they are following the CDM group protocol, they do not. Unfortunately, CDM members whose CD4 count first fell below 100 at week 48 do not have similar subjects that remain uncensored at week 96, as all LCM subjects whose CD4 first fell below 100 at week 48 were already censored at week 48. Thus, at week 96 we have a positivity violation even when we do not stratify on treatment group. When a positivity violation exists, it is not possible to consistently estimate survival under the strategy of interest. Thus, to consistently estimate survival under the strategy of interest requires that some subjects fail to comply with their assigned protocol.

As shown in Table 3, there was variability in compliance with the switching strategies considered here. In the DART Trial, the recommended CD4-based switching strategy in LCM changed from <50 to <100 in July 2006 and some discretion was allowed around switching for WHO 3 events. Additionally, some events managed as WHO stage 4 events by the trial clinicians were not subsequently adjudicated to have met predefined protocol criteria for a WHO stage 4 event (used here to define switching strategies).

To test the assumption that there was no direct effect of randomized group on mortality (as discussed above), we fitted a logistic regression model including randomized group and all baseline and time-dependent predictors of mortality in participants on first-line

ART. The odds ratio for CDM versus LCM was 0.79 (95% CI: 0.51, 1.24; P = 0.31), indicating no strong evidence against the "no direct effect" assumption. As there were participants compliant with the strategy "switch at first CD4 <50 or non-*Candida* WHO 4 event (CD4 <250)" in both randomized groups, we also fitted a joint model for this strategy. Weights were estimated separately by randomized group and randomized group was included in the outcome model. The odds ratio for CDM versus LCM was 0.82 (95% CI: 0.51, 1.34; P = 0.43); survival estimates were similar in both groups. This similarly indicates no strong evidence against the "no direct effect" assumption.

Because switching strategies in DART had differed between randomized groups, we anticipated that pooling randomized groups to estimate weights would improve the distribution of "switching weights." Web Table 1 shows the weight distribution estimated separately within randomized group and for pooled groups for different CD4 monitoring strategies. Under 12- or 24-weekly CD4 monitoring and estimating weights by group, there were some extreme weights in CDM, reflecting few participants switching following low CD4 without a protocol-accepted WHO 3/4 event; when pooling groups, weights were higher in the LCM group and the extreme weights were reduced (maximum: 797 versus 278). Mean weights were similar under both approaches, consistent with upweighting similar numbers of person-intervals compliant with the respective strategies. The pattern was less clear under no CD4 monitoring, although large weights in LCM were reduced by pooling groups.

Web Table 1. Distribution of Weights for Strategy Noncompliance* Under Different CD4 Monitoring Strategies for Switch at First CD4 <100 or Non-*Candida* WHO 4 (CD4 <250), DART Trial, Uganda and Zimbabwe, 2003–2008

Mean (Range) [1st and 99th Percentiles]	Weights Estimated Separately by Trial Randomization Group			Randomized Groups Combined Before Weight Estimation		
	Laboratory and Clinical Monitoring/ CD4 Monitoring Group (LCM)	Clinically Driven Monitoring/No CD4 Monitoring Group (CDM)	Both Randomization Groups	Laboratory and Clinical Monitoring/ CD4 Monitoring Group (LCM)	Clinically Driven Monitoring/No CD4 Monitoring Group (CDM)	Both Randomization Groups
CD4 at baseline[a] and every 12 weeks thereafter	1.12 (0.33, 37) [0.69, 4.71]	1.62 (0.33, 797) [0.67, 1.80]	1.37 (0.33, 797) [0.67, 2.83]	1.70 (0.33, 191) [0.68, 17]	1.08 (0.33, 278) [0.67, 1.24]	1.40 (0.33, 278) [0.67, 3.42]
CD4 at baseline[a] and every 24 weeks thereafter	1.16 (0.33, 45) [0.75, 5.30]	1.57 (0.33, 797) [0.67, 1.82]	1.36 (0.33, 797) [0.68, 3.50]	1.74 (0.33, 191) [0.74, 19]	1.08 (0.33, 278) [0.68, 1.32]	1.42 (0.33, 278) [0.70, 4.88]
CD4 at baseline[a] and every 48 weeks thereafter	1.22 (0.33, 449) [1.00, 5.01]	1.08 (0.33, 316) [0.67, 1.83]	1.15 (0.33, 449) [0.79, 3.27]	1.77 (0.33, 191) [1.00, 22]	1.07 (0.33, 278) [0.75, 1.87]	1.43 (0.33, 278) [0.88, 6.72]
CD4 at baseline[a]	1.25 (0.33, 449) [1.00, 3.38]	1.21 (0.33, 316) [1.00, 2.20]	1.23 (0.33, 449) [1.00, 2.66]	1.58 (0.33, 191) [1.00, 3.18]	1.18 (0.33, 706) [1.00, 2.94]	1.38 (0.33, 706) [1.00, 3.17]
No CD4 measurements	1.23 (0.33, 449) [1.00, 2.74]	1.20 (0.33, 316[b]) [1.00, 2.43]	1.21 (0.33, 449) [1.00, 2.58]	1.06 (0.34, 31) [1.00, 1.96]	1.22 (0.33, 706) [1.00, 3.71]	1.14 (0.33, 706) [1.00. 2.92]

* Weights shown here are to account for artificial censoring for noncompliance with the respective strategy only (i.e., they do not include the weights for loss to follow-up or the multiplier for randomization to STI).

[a] Baseline was the first 4-week visit at or after 48 consecutive weeks on first-line ART.

[b] Two individuals had person-months with weights >16; both individuals switched to second-line ART within 12 weeks of extra-pulmonary tuberculosis and had no other recent WHO 3/4 events. All other individuals had weights below 16 for all person-months.

WEB APPENDIX 2

Initially, 12 strategies ($X = 1, 2, \ldots 12$) were modelled. Ten strategies corresponded to “switch following first CD4 $<x$ or first non-*Candida* WHO stage 4 event,” with x set from 100 to 10 in 10-cells/mm^3 drops; $h(X)$ took values 10 to 1 and was included as a linear term, and $g(X)$ was set to 0. The eleventh strategy was “switch following two WHO 3 events or first non-*Candida* WHO 4,” $h(X)$ was set to 0, and $g(X)$ was set to level 1 of a categorical variable. The twelfth strategy was “switch following first non-*Candida* WHO 4,” $h(X)$ was set to 0, and $g(X)$ was set to level 2 of a categorical variable. In separate sensitivity analyses: i) $h(X)$ was allowed to be a cubic spline function and ii) only two CD4-based strategies were modelled: “switch following first CD4 <50 or first non-*Candida* WHO 4,” with $h(X)$ set to level 1 of a categorical variable ($g(X) = 0$), and “switch following first CD4 <100 or first non-*Candida* WHO 4,” with $h(X)$ set to level 2 of a categorical variable ($g(X) = 0$).

Web Figure 2. Percentage of follow-up spent by CD4 as observed in DART randomized groups and as estimated for different CD4 monitoring strategies under switch at first CD4 <100 or first non-*Candida* WHO 4 event (CD4 <250) using dynamic marginal structural models.

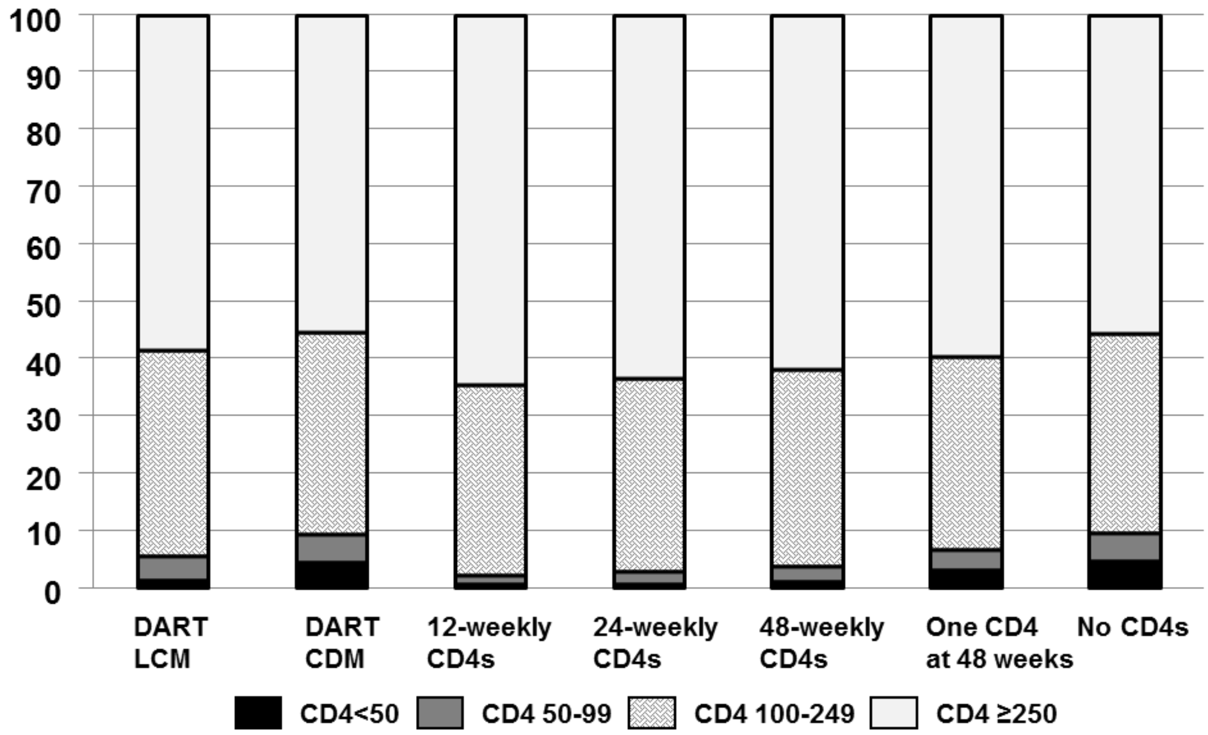

Percentage of follow-up with CD4<100							
CD4 50-99	4.31	4.91	1.67	2.07	2.70	3.51	4.80
CD4 <50	1.11	4.44	0.53	0.63	0.93	3.08	4.61

Web Table 2. Sensitivity Analyses: Estimated Survival Under Different Switching and CD4 Monitoring Strategies, DART Trial, Uganda and Zimbabwe, 2003–2008

	Survival (From Baseline = 48 Weeks on First-Line ART) (95% CI)										Proportion Alive and in Active Follow-up[a] (From Baseline = 48 Weeks on First-Line ART) (95% CI)	
	Switch at First CD4 <100 or Non-*Candida* WHO 4 (CD4 <250)		Switch at First CD4 <100 or Non-*Candida* WHO 4 (CD4 <250) Untruncated Weights		Switch at First CD4 <100 or Non-*Candida* or Non-TB WHO 4 (CD4 <250)		Switch at First CD4 <100 or Non-*Candida* WHO 4 (Irrespective of CD4 at Event)		Lagged Event History: Switch at First CD4 <100 or Non-*Candida* WHO 4 (CD4 <250)		Switch at First CD4 <100 or Non-*Candida* WHO 4 (CD4 <250)	
	192 Weeks	240 Weeks	192 Weeks	240 Weeks	192 Weeks	240 Weeks	192 Weeks	240 Weeks	192 Weeks	240 Weeks	192 Weeks	240 Weeks
CD4 at baseline[b] and every 12 weeks thereafter	0.97 (0.95, 0.98)	0.96 (0.94, 0.97)	0.97 (0.96, 0.98)	0.96 (0.95, 0.98)	0.96 (0.95, 0.98)	0.96 (0.94, 0.97)	0.95 (0.94, 0.97)	0.94 (0.92, 0.96)	0.96 (0.95, 0.98)	0.95 (0.94, 0.97)	0.91 (0.89, 0.93)	0.87 (0.84, 0.90)
CD4 at baseline[b] and every 24 weeks thereafter	0.97 (0.95, 0.98)	0.96 (0.95, 0.97)	0.97 (0.96, 0.98)	0.97 (0.95, 0.98)	0.97 (0.96, 0.98)	0.96 (0.95, 0.97)	0.95 (0.94, 0.97)	0.95 (0.93, 0.96)	0.96 (0.95, 0.97)	0.96 (0.94, 0.97)	0.91 (0.89, 0.93)	0.86 (0.83, 0.89)
CD4 at baseline[b] and every 48 weeks thereafter	0.96 (0.95, 0.97)	0.95 (0.93, 0.96)	0.97 (0.95, 0.98)	0.96 (0.94, 0.97)	0.96 (0.94, 0.97)	0.95 (0.93, 0.96)	0.95 (0.93, 0.96)	0.94 (0.92, 0.96)	0.96 (0.94, 0.97)	0.95 (0.93, 0.96)	0.91 (0.89, 0.93)	0.87 (0.84, 0.89)
CD4 at baseline[b]	0.96 (0.95, 0.97)	0.95 (0.93, 0.96)	0.97 (0.95, 0.97)	0.96 (0.95, 0.97)	0.95 (0.94, 0.96)	0.94 (0.92, 0.95)	0.95 (0.93, 0.96)	0.94 (0.92, 0.95)	0.96 (0.95, 0.97)	0.95 (0.93, 0.96)	0.91 (0.89, 0.92)	0.87 (0.84, 0.89)
No CD4 measurements	0.94 (0.92, 0.95)	0.92 (0.91, 0.94)	0.94 (0.92, 0.95)	0.92 (0.90, 0.94)	0.93 (0.92, 0.95)	0.91 (0.89, 0.93)	0.93 (0.91, 0.94)	0.91 (0.89, 0.93)	0.94 (0.93, 0.95)	0.93 (0.91, 0.94)	0.89 (0.88, 0.91)	0.85 (0.82, 0.87)
Survival gain: 12-weekly CD4s versus no CD4 monitoring	2.8 (1.3, 4.4)	3.5 (1.4, 5.6)	3.5 (1.7, 5.3)	4.2 (1.8, 6.8)	3.4 (1.9, 5.0)	4.2 (2.1, 6.6)	2.5 (1.1, 4.0)	3.1 (1.1, 5.1)	2.0 (0.5, 3.2)	2.5 (0.4, 4.4)	1.9 (0.1, 3.6)	2.4 (-0.7, 5.3)

(Continued)

Survival gain: 12-weekly CD4s versus 24-weekly CD4s	-0.1 (-0.8, 0.5)	-0.2 (-1.4, 0.7)	-0.1 (-0.8, 0.4)	-0.2 (-1.2, 0.5)	-0.1 (-0.8, 0.5)	-0.2 (-1.3, 0.6)	-0.1 (-0.9, 0.5)	-0.2 (1.3, 0.6)	-0.1 (-0.8, 0.5)	-0.1 (-1.3, 0.7)	0.5 (-0.5, 1.4)	0.8 (-0.8, 2.5)
Survival gain: 12-weekly CD4s versus CD4 at baseline[b]	0.7 (-0.6, 1.8)	0.9 (-1.0, 2.7)	0.5 (-0.5, 1.4)	0.6 (-0.7, 1.9)	1.4 (0.1, 2.8)	1.8 (-0.1, 3.9)	0.6 (-0.5, 1.7)	0.8 (-0.7, 2.3)	0.5 (-0.6, 1.4)	0.7 (-0.9, 2.1)	0.4 (-1.2, 1.9)	0.5 (-2.0, 3.1)
Survival gain: CD4 at baseline[b] versus no CD4 monitoring	2.0 (1.1, 3.0)	2.4 (1.3, 3.9)	2.9 (1.7, 4.7)	3.6 (2.0, 5.9)	2.0 (1.1, 2.9)	2.4 (1.3, 3.6)	1.9 (1.1, 2.8)	2.3 (1.3, 3.5)	1.4 (0.7, 2.3)	1.8 (0.9, 3.1)	1.5 (0.5, 2.4)	1.9 (0.3, 3.3)

ART, antiretroviral therapy; CI, confidence interval; WHO, World Health Organization.

[a] Combined event of "death or loss to follow-up."

[b] Baseline was the first 4 week visit at or after 48 consecutive weeks on first-line ART.

Survival gains are percentage gains.

WEB APPENDIX 3

Survival Estimates by Use of Cotrimoxazole in Weeks 48–72 on ART

In the DART Trial, we demonstrated a benefit for current use of cotrimoxazole (CTX) in the first 72 weeks on ART. No benefit was shown for past use or for use after 72 weeks on ART (1). Since baseline for this analysis was 48 weeks on ART, any benefit of CTX would be restricted to the first 24 weeks of follow-up, but if prescription of CTX differed substantially between randomized groups, this could have influenced our results (see Web Appendix 1, question 1). Here we estimate survival under different monitoring strategies for those on and off CTX. Analysis was restricted to 1442 individuals in LCM and 1450 individuals in CDM who completed 48 consecutive weeks on first-line ART at 48 weeks from ART initiation (Web Table 3). 615/1442 (43%) in LCM and 755/1450 (48%) participants in CDM were on CTX at 48 weeks ($P = 0.004$). Time-independent weights were estimated for use of CTX at 48 weeks; predictors included combinations of center and first-line ART regimen; and at 48 weeks: CD4, body mass index, and hemoglobin; WHO 4 event 24–48 weeks after starting ART and TB 24–48 weeks after starting ART. Individuals were artificially censored if they started CTX ($n = 149$ in LCM; $n = 121$ in CDM) or stopped CTX ($n = 54$ in LCM; $n = 34$ in CDM) before 72 weeks on ART. Weights were estimated for this new censoring and predictors included: center, use of second-line ART in the previous interval, and time-updated body mass index, hemoglobin, CD4 (<100, ≥100), and history of WHO 3/4 events; and interaction terms for time-updated factors and on/off CTX at 48 weeks. After 72 weeks on ART, weights for remaining uncensored up to 72 weeks were carried forward. Switching and loss-to-follow-up weights were re-estimated in intervals prior to censoring for starting/stopping

CTX, and models included use of CTX at 48 weeks but were otherwise as described in the Methods. The product of weights for CTX at 48 weeks (baseline) and time-updated switching, substudy, censoring due to loss to follow-up, and censoring due to change in CTX use was used to weight outcome models. Weights were truncated at 10 as previously.

Survival was similar at 192 and 240 weeks in patients on or off CTX in weeks 48–72 on ART, and survival gains for more frequent CD4 monitoring were similar regardless of whether CTX was or was not prescribed.

Web Table 3. Estimated Survival Under Different CD4 Monitoring Strategies by Use of Cotrimoxazole Prophylaxis, DART Trial, Uganda and Zimbabwe, 2003–2008

	On Cotrimoxazole for Weeks 48–72 on ART			Off Cotrimoxazole for Weeks 48–72 on ART		
	48 Weeks	192 Weeks	240 Weeks	48 Weeks	192 Weeks	240 Weeks
CD4 at baseline[a] and every 12 weeks thereafter	0.99 (0.99, 1.0)	0.97 (0.95, 0.98)	0.96 (0.93, 0.98)	0.99 (0.98, 0.99)	0.97 (0.95, 0.98)	0.96 (0.95, 0.98)
CD4 at baseline[a] and every 24 weeks thereafter	0.99 (0.99, 1.0)	0.97 (0.95, 0.98)	0.96 (0.94, 0.98)	0.99 (0.98, 0.99)	0.97 (0.96, 0.98)	0.96 (0.95, 0.98)
CD4 at baseline[a] and every 48 weeks thereafter	0.99 (0.98, 0.99)	0.96 (0.94, 0.97)	0.95 (0.93, 0.97)	0.99 (0.98, 0.99)	0.96 (0.95, 0.98)	0.96 (0.94, 0.97)
CD4 at baseline[a]	0.99 (0.98, 0.99)	0.95 (0.94, 0.97)	0.94 (0.92, 0.96)	0.99 (0.98, 0.99)	0.96 (0.94, 0.97)	0.95 (0.93, 0.97)
No CD4 measurements	0.98 (0.97, 0.99)	0.93 (0.91, 0.95)	0.92 (0.89, 0.94)	0.98 (0.97, 0.99)	0.94 (0.92, 0.96)	0.93 (0.91, 0.95)
Survival gain: 12-weekly CD4s versus no CD4 monitoring	0.7 (0.3, 1.3)	3.3 (1.6, 5.1)	3.8 (1.6, 6.3)	0.8 (0.3, 1.5)	2.7 (1.1, 4.4)	3.2 (1.3, 5.3)
Survival gain: 12-weekly CD4s versus 24-weekly CD4s	0.0 (0.0, 0.2)	-0.1 (-1.1, 0.4)	-0.3 (-1.7, 0.4)	0.0 (0.0, 0.1)	-0.2 (-0.9, 0.2)	-0.3 (-1.3, 0.2)
Survival gain: 12-weekly CD4s versus CD4 at baseline[a]	0.2 (0.1, 0.5)	1.3 (-0.2, 2.5)	1.5 (-0.1, 3.2)	0.1 (0.0, 0.5)	0.9 (-0.1, 2.2)	1.1 (-0.3, 2.9)
Survival gain: CD4 at baseline[a] versus no CD4 monitoring	0.5 (0.2, 1.0)	2.0 (0.9, 3.5)	2.4 (1.0, 4.3)	0.6 (0.2, 1.2)	1.8 (0.9, 2.7)	2.0 (1.0, 3.2)

[a] Baseline was the first 4-week visit at 48 consecutive weeks on first-line ART.

Survival gains are percentage gains.

Reference

1. Walker AS, Ford D, Gilks CF, et al. Daily co-trimoxazole prophylaxis in severely immunosuppressed HIV-infected adults in Africa started on combination antiretroviral therapy: an observational analysis of the DART cohort. *Lancet* 2010;375(9722):1278–1286.
